# Supplementary material for: Decreasing Trends of Secondary Primary Colorectal Cancer among Women with Uterine Cancer: A Population-Based Analysis
Source: J Clin Med. 2019 May 20;8(5):714. doi: 10.3390/jcm8050714 (PMC6571981; doi:10.3390/jcm8050714)
Supplement: Supplementary file 1 [file jcm-08-00714-s001.pdf]

Decreasing trends of secondary primary colorectal cancer among women with uterine cancer: a population-based analysis

Koji Matsuo, MD, PhD[1,2,*]; Rachel S. Mandelbaum, MD[1]; Hiroko Machida, MD, PhD[3]; Kosuke Yoshihara, MD, PhD[4]; Franco M. Muggia, MD[5]; Lynda D. Roman, MD[1,2]; Jason D. Wright, MD[6]

1) Division of Gynecologic Oncology, Department of Obstetrics and Gynecology, University of Southern California, Los Angeles, CA, USA.

2) Norris Comprehensive Cancer Center, University of Southern California, Los Angeles, CA, USA.

3) Department of Obstetrics and Gynecology, Tokai University School of Medicine, Kanagawa, Japan.

4) Department of Obstetrics and Gynecology, Niigata University School of Medicine, Niigata, Japan

5) Division of Medical Oncology, Department of Medicine, New York University, New York, New York, USA

6) Division of Gynecologic Oncology, Department of Obstetrics and Gynecology, Columbia University College of Physicians and Surgeons, New York, NY, USA.

[*]**Address correspondence to:**
Koji Matsuo, MD, PhD, Division of Gynecologic Oncology, Department of Obstetrics and Gynecology, University of Southern California, 2020 Zonal Avenue, IRD 520, Los Angeles, CA 90033, USA, Tel: +1-323-409-3416, FAX: +1-323-206-3427, Email: koji.matsuo@med.usc.edu

Keywords: colorectal cancer; uterine cancer; secondary cancer; trend; survival.

Supplemental Figure S1. CONSORT diagram for study schema.

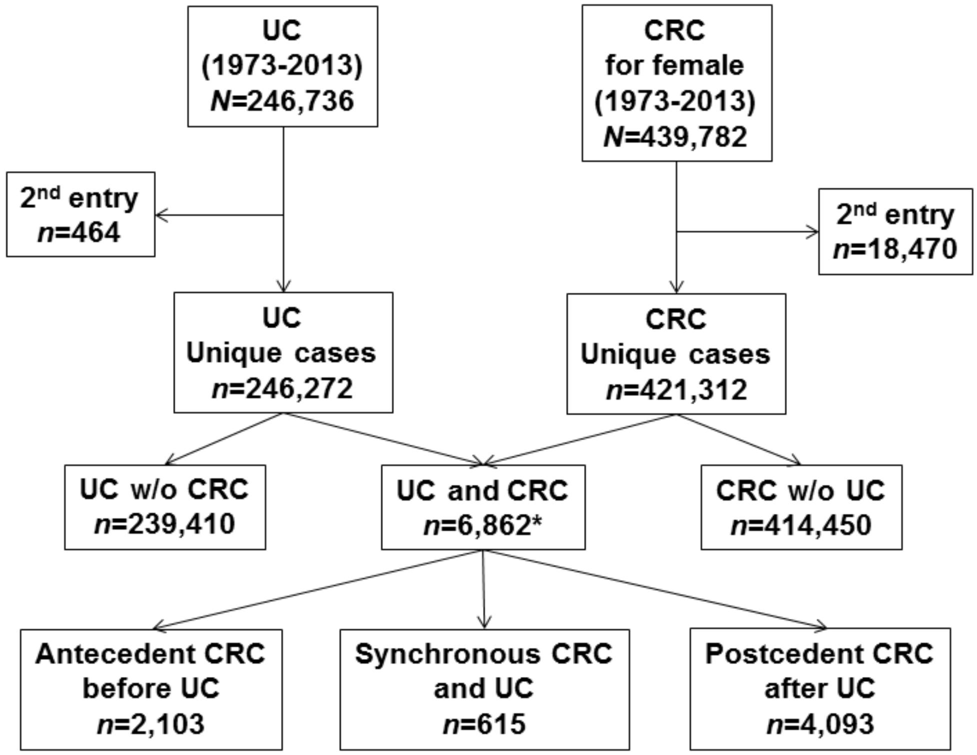

*51 cases with lack of month information were not assessable for relative time interval between the two cancer diagnoses. Abbreviations: UC, uterine cancer; CRC, colorectal cancer; and w/o without.

Supplemental Figure S2. Median follow-up time among cancer survivors.

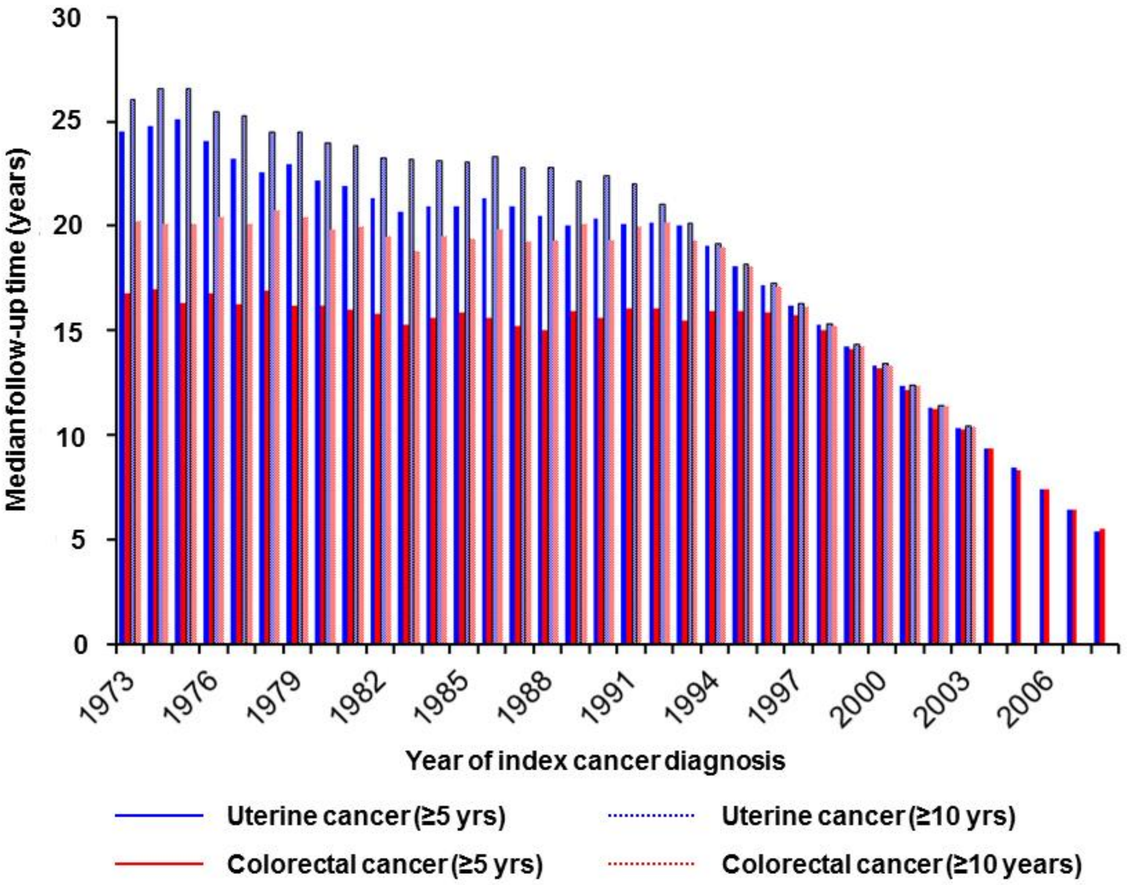

Among ≥5 years cohort for uterine cancer, the median follow-up time was 11.7 (IQR 8-17.9) years. Among ≥10 years cohort for uterine cancer, the median follow-up time was 16.0 (IQR 12.4-22.1) years. Among ≥5 years cohort for colorectal cancer, the median follow-up time was 10.3 (IQR 7.3-14.3) years. Among ≥10 years cohort for colorectal cancer, the median follow-up time was 14.1 (IQR 11.8-19.1) years. Abbreviations: yrs, years; and IQR, interquartile range.

Supplemental Figure S3. Trends of postcedent secondary primary cancer among cancer survivors per year stratified chronology.

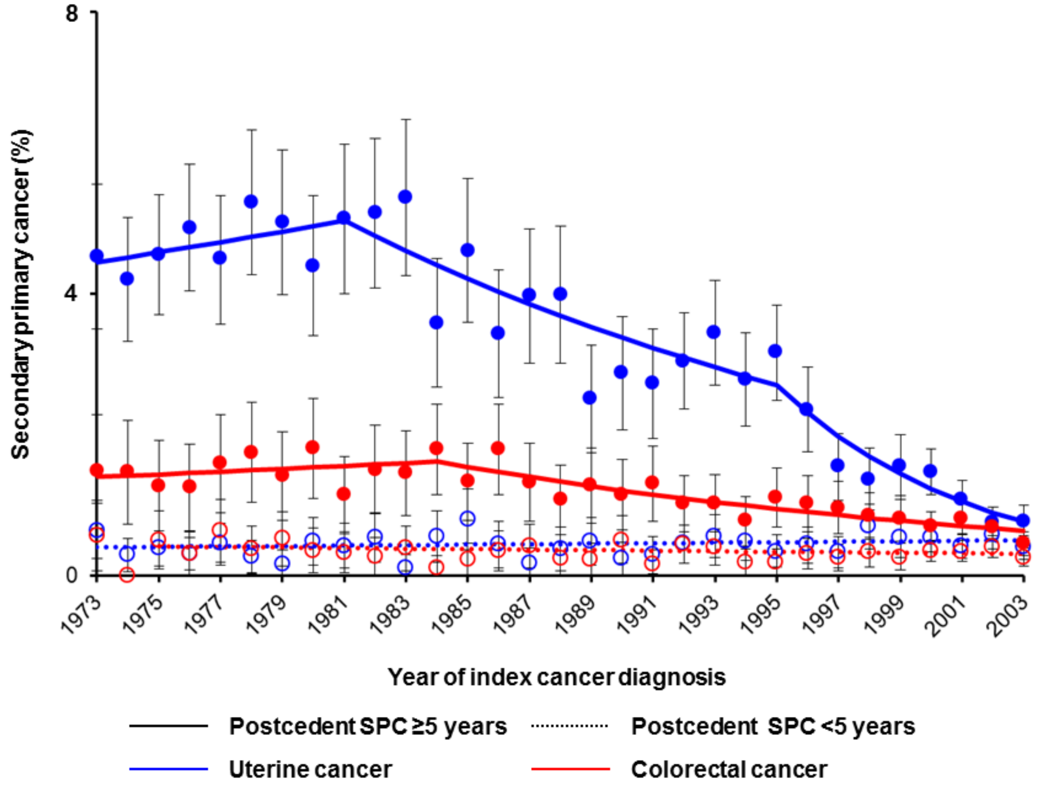

Results of Figure 3A (follow-up ≥10 years) were further stratified based on the timing of postcedent secondary cancer diagnosis: ≥5 years (solid lines) *versus* <5 years (dashed lines) from the index cancer diagnosis. Raw results of temporal trends are shown in Supplemental Table S7.

Supplemental Figure S4. Trends in secondary primary cancer per age.

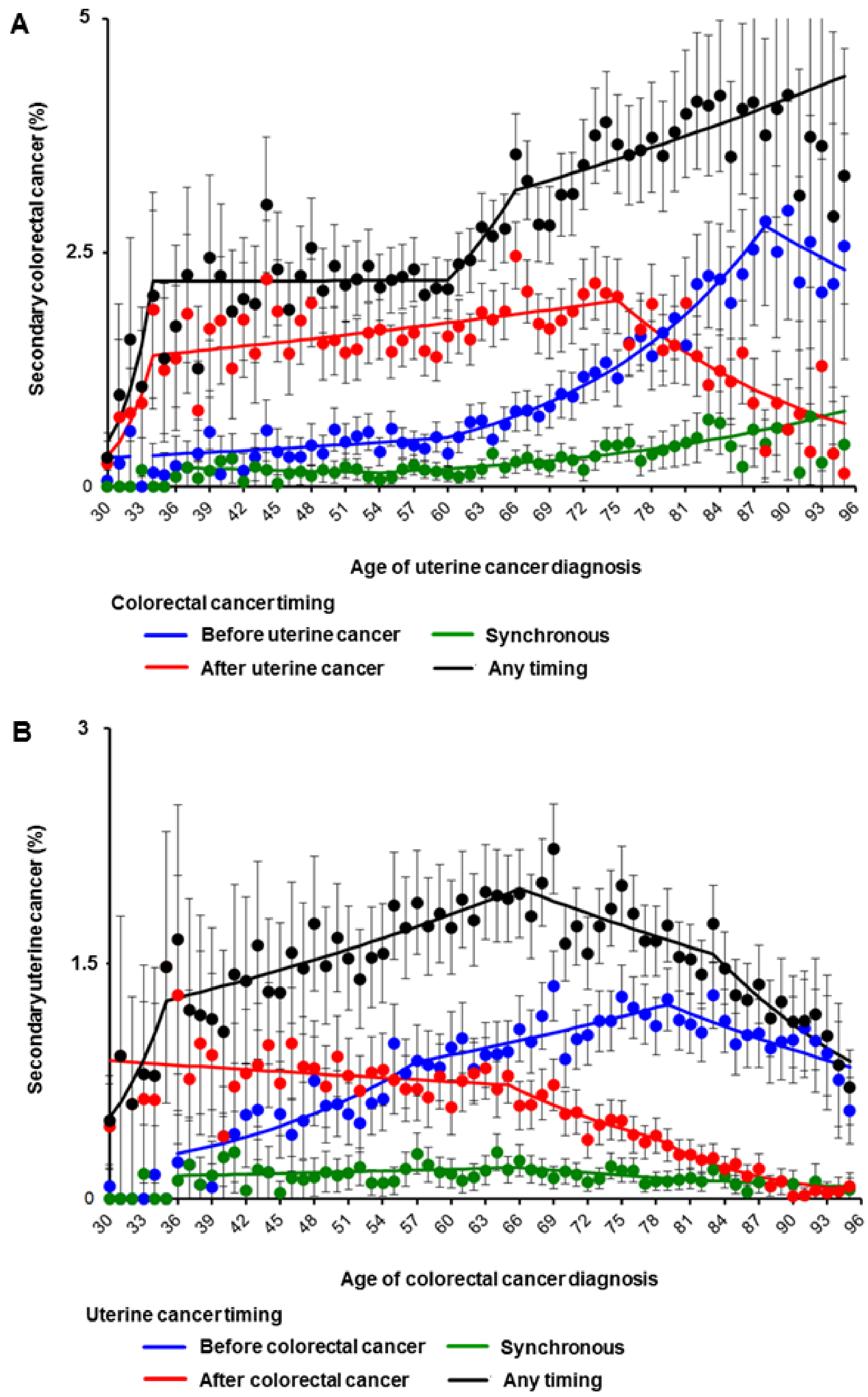

(A) Proportion of uterine cancer women who had secondary colorectal cancer is shown per age. **(B)** Proportion of colorectal cancer women who had secondary uterine cancer is shown per age. Dots represent actual observed values. Bars represent standard confidence intervals. Colored lines represent modeled lines. Raw results of temporal trends are shown in Supplemental Table S8.

Supplemental Figure S5. Absolute time interval between the two cancer diagnoses per age at the index cancer diagnosis.

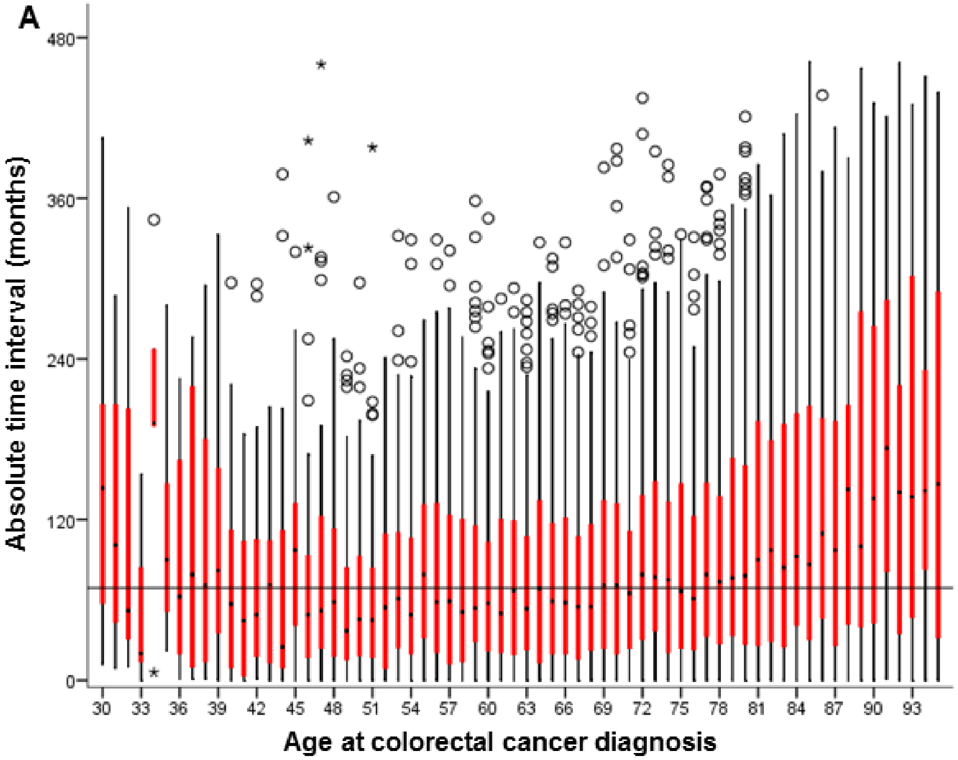

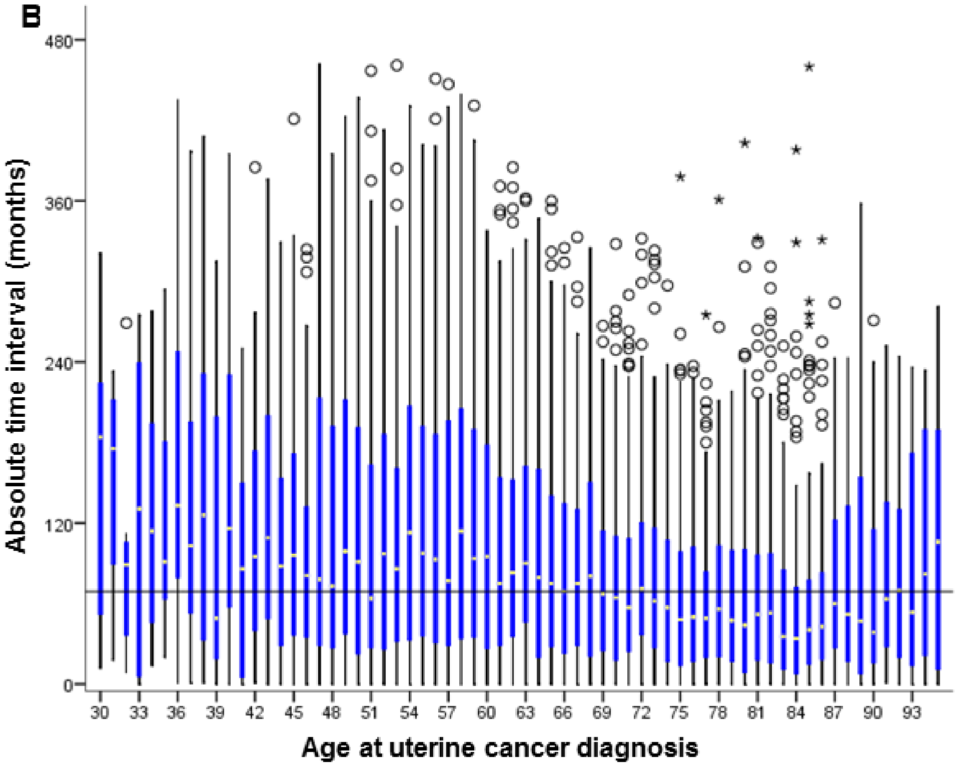

Among 6,811 women who had the two cancer diagnoses, absolute time interval between the two diagnoses is displayed per age at the index cancer diagnosis. **(A)** Absolute time interval to uterine cancer diagnosis is shown per age of colorectal cancer diagnosis. **(B)** Absolute time interval to colorectal cancer diagnosis is shown per age of uterine cancer diagnosis. The horizontal line shows median value of the absolute time interval (5.8 years).

Supplemental Figure S6. Secondary primary cancer-specific survival among survivors.

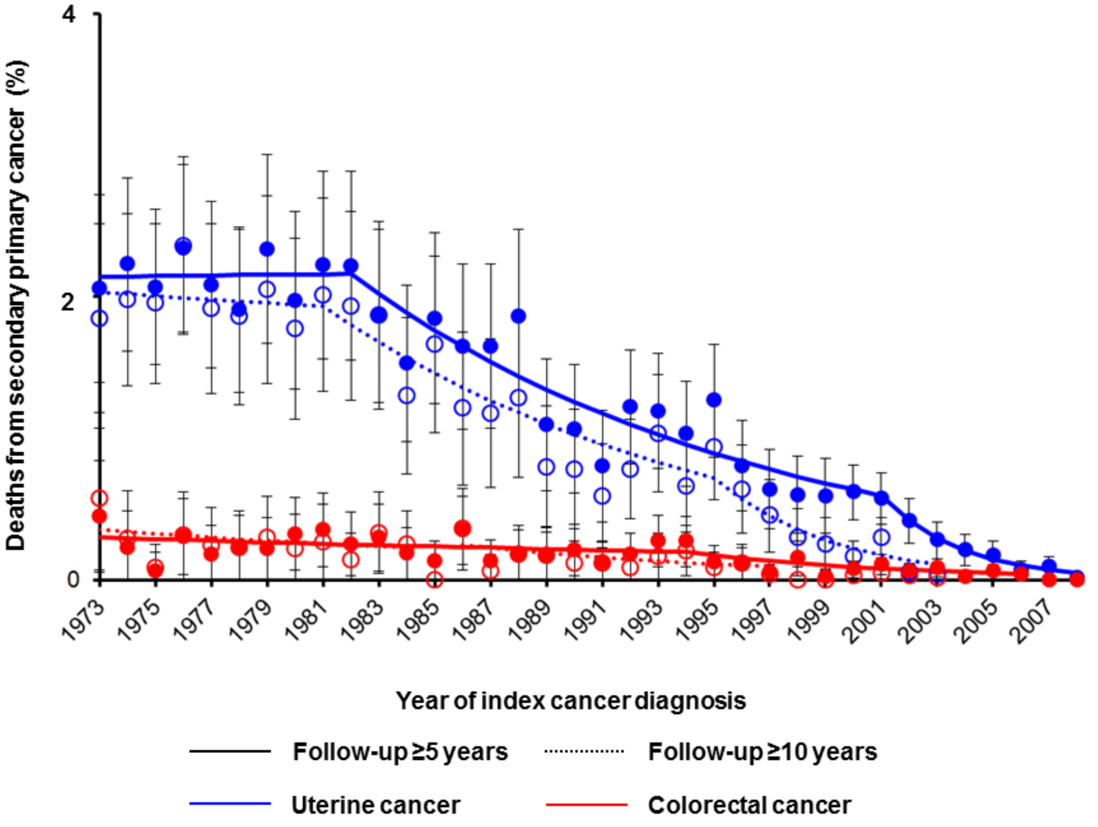

The number of cancer survivors who died of secondary primary cancer per year of index cancer diagnosis is shown for **(A)** secondary colorectal cancer deaths among uterine cancer survivors and **(B)** secondary uterine cancer deaths among colorectal cancer survivors. Raw results of temporal trends are shown in Supplemental Table S9.

Supplemental Figure S7. Secondary colorectal cancer among endometrial cancer.

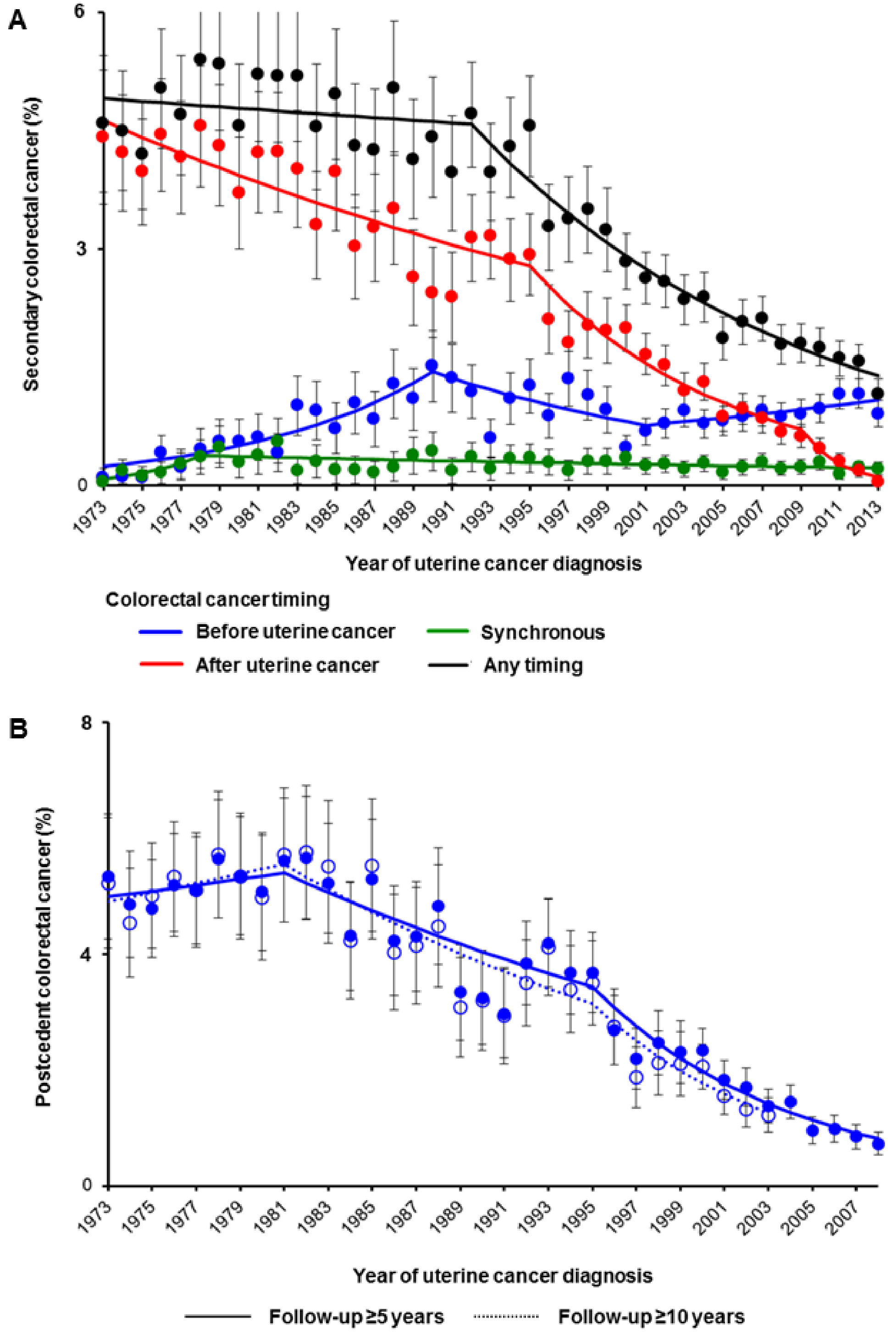

(A) Temporal trends are shown for secondary colorectal cancer among endometrial cancer based on the timing of secondary colorectal cancer. **(B)** Among women with endometrial cancer who did not die of disease and had follow-up ≥5 years (solid lines) or ≥10 years (dashed lines), temporal trends of the

incidence of postcedent colorectal cancer were examined. Dots represent actual observed values. Bars represent standard confidence intervals. Colored lines represent modeled lines. Raw results of temporal trends are shown in Supplemental Table S10-11.

Supplemental Figure S8. Secondary colorectal cancer among endometrioid type endometrial cancer.

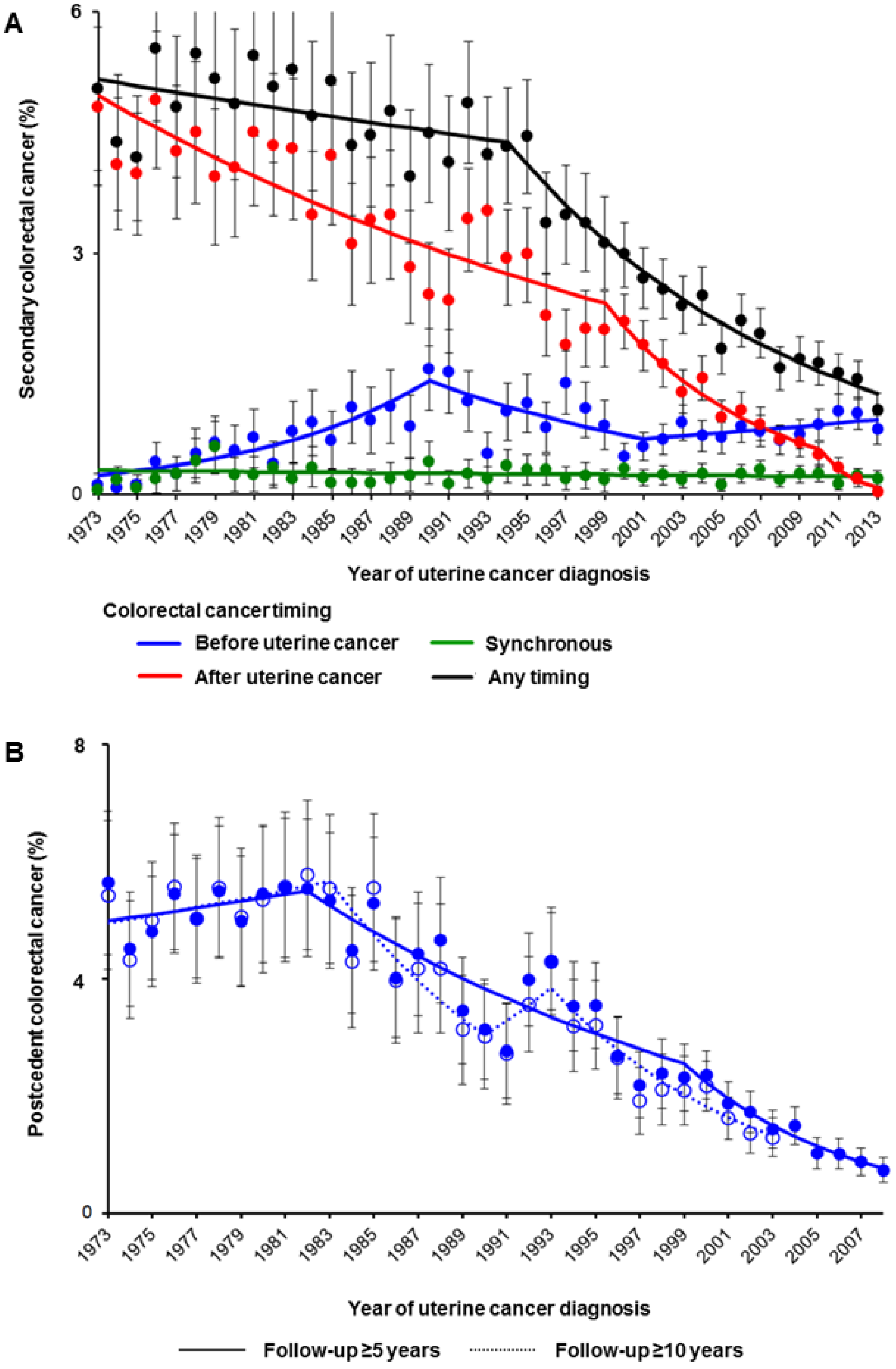

(A) Temporal trends are shown for secondary colorectal cancer among endometrioid type endometrial cancer based on the timing of secondary colorectal cancer. **(B)** Among women with endometrioid type

endometrial cancer who did not died of disease and had follow-up ≥5 years (solid lines) or ≥10 years (dashed lines), temporal trends of the incidence of postcedent colorectal cancer were examined. Dots represent actual observed values. Bars represent standard confidence intervals. Colored lines represent modeled lines. Raw results of temporal trends are shown in Supplemental Table S12-13.

Supplemental Table S1. Patient demographics (endometrial cancer cohort).

Characteristic	No CRC	Antecedent-CRC	Synchronous-CRC	Postcedent-CRC	*P*-value
Number	*n*=220,741	*n*=1,981	*n*=571	*n*=3,944	
Age	63.1 (±12.3)	70.3 (±12.3)	68.4 (±12.3)	63.2 (±11.4)	**<0.001**
Year					**<0.001**
Before 1980	19,073 (8.6%)	56 (2.8%)	46 (8.1%)	859 (21.8%)	
1980-9	25,167 (11.4%)	224 (11.3%)	76 (13.3%)	949 (24.1%)	
1990-9	36,708 (16.6%)	426 (21.5%)	112 (19.6%)	943 (23.9%)	
2000-9	92,536 (41.9%)	771 (38.9%)	232 (40.6%)	1,076 (17.3%)	
2010 or later	47,257 (21.4%)	504 (25.4%)	105 (18.4%)	117 (3.0%)	
Race/ethnicity					**<0.001**
White	171,885 (77.9%)	1,567 (79.1%)	460 (80.6%)	3,333 (84.5%)	
Black	16,027 (7.3%)	141 (7.1%)	57 (10.0%)	220 (5.6%)	
Hispanic	17,018 (7.7%)	129 (6.5%)	30 (5.3%)	162 (4.1%)	
Asian	11,541 (5.2%)	114 (5.8%)	19 (3.3%)	182 (4.6%)	
Others*	4,270 (1.9%)	30 (1.5%)	5 (0.9%)	47 (1.2%)	
Area					**<0.001**
West	113,719 (51.5%)	958 (48.4%)	244 (42.7%)	1,903 (48.3%)	
Central	51,448 (23.3%)	543 (27.4%)	157 (27.5%)	1,149 (29.1%)	
East	55,574 (25.2%)	480 (24.2%)	170 (29.8%)	892 (22.6%)	
Marital status					**<0.001**
Single	32,058 (14.5%)	181 (9.1%)	81 (14.2%)	454 (11.5%)	
Married	115,301 (52.2%)	900 (45.4%)	275 (48.2%)	2,223 (56.4%)	
Others*	73,382 (33.2%)	900 (45.4%)	215 (37.7%)	1,267 (32.1%)	
Histology types					**<0.001**
Endometrioid	172,784 (78.3%)	1,416 (71.5%)	416 (72.9%)	3,250 (82.4%)	
Serous	13,975 (6.3%)	173 (8.7%)	55 (9.6%)	221 (5.6%)	
Clear cell	2,936 (1.3%)	45 (2.3%)	13 (2.3%)	41 (1.0%)	
Carcinosarcoma	10,664 (4.8%)	159 (8.0%)	28 (4.9%)	84 (2.1%)	
Mixed	7,318 (3.3%)	95 (4.8%)	17 (3.0%)	45 (1.1%)	
Others	13,064 (5.9%)	93 (4.7%)	42 (7.4%)	303 (7.7%)	
Stage					**<0.001**
I	144,074 (65.3%)	1,223 (61.7%)	312 (54.6%)	2,443 (61.9%)	
II	9,672 (4.4%)	115 (5.8%)	32 (5.6%)	167 (4.2%)	
III	19,235 (8.7%)	213 (10.8%)	62 (10.9%)	213 (5.4%)	
IV	15,320 (6.9%)	147 (7.4%)	61 (10.7%)	104 (2.6%)	
Unknown	32,440 (14.7%)	283 (14.3%)	104 (18.2%)	1,017 (25.8%)	
Grade					**<0.001**
1	81,211 (36.8%)	528 (26.7%)	168 (29.4%)	1,486 (37.7%)	
2	59,830 (27.1%)	552 (27.9%)	160 (28.0%)	1,280 (32.5%)	
3	46,449 (21.0%)	569 (28.7%)	141 (24.7%)	635 (16.1%)	
Unknown	33,251 (15.1%)	332 (16.8%)	102 (17.9%)	543 (13.8%)	
Tumor size					**<0.001**
≤2 cm	21,049 (9.5%)	198 (10.0%)	46 (8.1%)	271 (6.9%)	
>2 cm	73,887 (33.5%)	702 (35.4%)	170 (29.8%)	846 (21.5%)	
Unknown	125,805 (57.0%)	1,081 (54.6%)	355 (62.2%)	2,827 (71.7%)	
Pelvic lymph node[†]					**<0.001**
Not involved	177,567 (92.4%)	1,528 (92.3%)	417 (92.1%)	2,833 (96.0%)	
Involved	14,562 (7.6%)	128 (7.7%)	36 (7.9%)	117 (4.0%)	
Lymph node ratio (%)[†]	20.0 (9.1-50.0)	30.8 (12.5-56.3)	29.4 (16.7-100)	18.6 (10.0-38.2)	**0.008**
Hysterectomy					**<0.001**
No	15,747 (7.1%)	260 (13.1%)	53 (9.3%)	90 (2.3%)	
Yes	176,095 (79.8%)	1,596 (80.6%)	427 (74.8%)	2,644 (67.0%)	
Unknown	28,899 (13.1%)	125 (6.3%)	91 (15.9%)	1,210 (30.7%)	
Radiotherapy					**<0.001**
None	151,650 (68.7%)	1,444 (72.9%)	435 (76.2%)	2,373 (60.2%)	
External beam	45,860 (20.8%)	340 (17.2%)	105 (18.4%)	1,092 (27.7%)	

Implants	12,860 (5.8%)	128 (6.5%)	19 (3.3%)	136 (3.4%)
Both	6,632 (3.0%)	31 (1.6%)	5 (0.9%)	285 (7.2%)
Unknown	3,739 (1.7%)	38 (1.9%)	7 (1.2%)	58 (1.5%)

Number (percent per column), median (interquartile range), or mean (standard deviation) is shown. Cases without known time interval to secondary colorectal cancer were not included. Significant *P*-values are emboldened. *including unknown cases. †among staged cases with available results. Abbreviation: CRC, secondary colorectal cancer.

Supplemental Table S2. Patient demographics for endometrioid type endometrial cancer based on timing of secondary colorectal cancer.

Characteristic	No CRC	Antecedent-CRC	Synchronous-CRC	Postcedent-CRC	*P*-value
Number	*n*=172,784	*n*=1,416	*n*=416	*n*=3,250	
Age	62.3 (±12.2)	69.4 (±12.7)	68.2 (±12.4)	63.1 (±11.4)	**<0.001**
Year					**<0.001**
Before 1980	14,821 (8.6%)	47 (3.3%)	39 (9.4%)	679 (20.9%)	
1980-9	19,422 (11.2%)	162 (11.4%)	46 (11.1%)	769 (23.7%)	
1990-9	29,267 (16.9%)	330 (23.3%)	78 (18.8%)	788 (24.2%)	
2000-9	73,062 (42.3%)	535 (37.8%)	177 (42.5%)	918 (28.2%)	
2010 or later	36,212 (21.0%)	342 (24.2%)	76 (18.3%)	96 (3.0%)	
Race/ethnicity					**<0.001**
White	137,422 (79.5%)	1,143 (80.7%)	344 (82.7%)	2,767 (85.1%)	
Black	9,801 (5.7%)	84 (5.9%)	36 (8.7%)	169 (5.2%)	
Hispanic	13,090 (7.6%)	88 (6.2%)	23 (5.5%)	135 (4.2%)	
Asian	8,980 (5.2%)	77 (5.4%)	8 (1.9%)	140 (4.3%)	
Others*	3,491 (2.0%)	24 (1.7%)	5 (1.2%)	39 (1.2%)	
Area					**<0.001**
West	89,070 (51.5%)	673 (47.5%)	176 (42.3%)	1,559 (48.0%)	
Central	40,364 (23.4%)	405 (28.6%)	118 (28.4%)	949 (29.2%)	
East	43,350 (25.1%)	338 (23.9%)	122 (29.3%)	742 (22.8%)	
Marital status					**<0.001**
Single	25,190 (14.6%)	143 (10.1%)	66 (15.9%)	367 (11.3%)	
Married	92,356 (53.5%)	655 (46.3%)	199 (47.8%)	1,840 (56.6%)	
Others*	55,238 (32.0%)	618 (43.6%)	151 (36.3%)	1,043 (32.1%)	
Stage					**<0.001**
I	122,613 (71.0%)	942 (66.5%)	256 (61.5%)	2,107 (64.8%)	
II	7,150 (4.1%)	71 (5.0%)	19 (4.6%)	132 (4.1%)	
III	11,799 (6.8%)	118 (8.3%)	38 (9.1%)	160 (4.9%)	
IV	7,481 (4.3%)	68 (4.8%)	29 (7.0%)	63 (1.9%)	
Unknown	23,741 (13.7%)	217 (15.3%)	74 (17.8%)	788 (24.2%)	
Grade					**<0.001**
1	74,723 (43.2%)	486 (34.3%)	158 (38.0%)	1,357 (41.8%)	
2	51,960 (30.1%)	474 (33.5%)	129 (31.0%)	1,112 (34.2%)	
3	25,804 (14.9%)	270 (19.1%)	72 (17.3%)	419 (12.9%)	
Unknown	20,297 (11.7%)	186 (13.1%)	57 (13.7%)	362 (11.1%)	
Tumor size					**<0.001**
≤2 cm	17,671 (10.2%)	150 (10.6%)	35 (8.4%)	239 (7.4%)	
>2 cm	54,554 (31.6%)	442 (31.2%)	117 (28.1%)	675 (20.8%)	
Unknown	100,559 (58.2%)	824 (58.2%)	264 (63.5%)	2,336 (71.9%)	
Pelvic lymph node†					**<0.001**
Not involved	177,567 (95.7%)	1,528 (95.7%)	417 (94.8%)	2,833 (97.3%)	
Involved	7,946 (4.3%)	69 (4.3%)	23 (5.2%)	78 (2.7%)	
Lymph node ratio (%)†	16.7 (8.1-40.0)	23.0 (10.0-53.4)	29.4 (16.7-100)	13.4 (9.3-25.0)	**0.036**
Hysterectomy					**<0.001**
No	11,489 (6.6%)	190 (13.4%)	42 (10.1%)	76 (2.3%)	
Yes	139,191 (80.6%)	1,127 (79.6%)	309 (74.3%)	2,215 (68.2%)	
Unknown	22,104 (12.8%)	99 (7.0%)	65 (15.6%)	959 (29.5%)	
Radiotherapy					**<0.001**
None	122,788 (71.1%)	1,070 (75.6%)	326 (78.4%)	2,007 (61.8%)	
External beam	32,440 (18.8%)	222 (15.7%)	68 (16.3%)	844 (26.0%)	
Implants	9,822 (5.7%)	80 (5.6%)	15 (3.6%)	126 (3.9%)	
Both	5,110 (3.0%)	22 (1.6%)	4 (1.0%)	228 (7.0%)	
Unknown	2,624 (1.5%)	22 (1.6%)	3 (0.7%)	45 (1.4%)	

Number (percent per column), median (interquartile range), or mean (standard deviation) is shown. Significant *P*-values are emboldened. Cases without known time interval to secondary colorectal cancer were not included. *including unknown cases. †among staged cases with available results. Abbreviation: CRC, secondary colorectal cancer.

Supplemental Table S3. Temporal trends of age at the index cancer diagnosis based on presence of secondary primary cancer (for Figure 1A-B).

Cohort	SPC	Segment	Lower Endpoint	Upper Endpoint	APC	Lower CI	Upper CI	Test Statistic	*P*-value
Uterine	Yes	1	1973	1991	0.7	0.5	0.8	8.7	**<0.001**
		2	1991	2013	0.0	-0.1	0.1	0.7	0.48
	No	1	1973	1983	0.6	0.5	0.7	14.4	**<0.001**
		2	1983	1992	0.1	0	0.2	1.5	0.14
		3	1992	2007	-0.3	-0.3	-0.3	-15.2	**<0.001**
		4	2007	2013	0.1	0	0.2	1.4	0.18
Colorectal	Yes	1	1973	1994	0.6	0.5	0.7	9.1	**<0.001**
		2	1994	2013	-0.1	-0.2	0.0	-2.2	**0.032**
	No	1	1973	1986	0.3	0.3	0.3	14.8	**<0.001**
		2	1986	1998	0.0	-0.1	0.0	-0.7	0.51
		3	1998	2013	-0.4	-0.4	-0.3	-33.6	**<0.001**

Significant *P*-values are emboldened. Abbreviations: SPC, secondary primary cancer; APC, annual rate of percent change, and CI, confidence interval.

Supplemental Table S4. Temporal trends for secondary primary cancer per year (for Figure 2A-B).

Cohort	SPC Timing	Segment	Lower Endpoint	Upper Endpoint	APC	Lower CI	Upper CI	Test Statistic	*P*-value
Uterine	Any timing	1	1973	1992	-0.4	-1.2	0.4	-0.9	0.36
		2	1992	2013	-5.4	-6.0	-4.9	-19	**<0.001**
	Antecedent	1	1973	1990	10.7	6.1	15.5	4.9	**<0.001**
		2	1990	2001	-5.6	-10.3	-0.6	-2.3	**0.029**
		3	2001	2013	3.2	0.7	5.9	2.6	**0.014**
	Synchronous	1	1973	1979	23.7	1.1	51.3	2.1	**0.039**
		2	1979	2013	-1.6	-2.5	-0.8	-3.7	**<0.001**
	Postcedent	1	1973	1995	-2.2	-3	-1.5	-6	**<0.001**
		2	1995	2009	-9.3	-11.1	-7.6	-10.4	**<0.001**
		3	2009	2013	-38.1	-52	-20.3	-3.9	**<0.001**
Colorectal	Any timing	1	1973	1986	5.7	3.6	8.0	5.5	**<0.001**
		2	1986	1998	0.4	-1.3	2.2	0.5	0.64
		3	1998	2001	-10.6	-27.3	9.9	-1.1	0.28
		4	2001	2013	0.2	-1.0	1.3	0.3	0.79
	Antecedent	1	1973	1986	13	8.4	17.8	6	**<0.001**
		2	1986	1998	1.7	-0.8	4.2	1.4	0.17
		3	1998	2001	-16.2	-37.5	12.3	-1.2	0.23
		4	2001	2013	4.2	2.8	5.8	5.9	**<0.001**
	Synchronous	1	1973	2013	-0.2	-1.0	0.6	-0.5	0.60
	Postcedent	1	1973	2001	-0.7	-1.3	-0.1	-2.5	**0.016**
		2	2001	2011	-9.5	-11.8	-7.0	-7.7	**<0.001**
		3	2011	2013	-63.2	-85.8	-4.7	-2.1	**0.04**

Significant *P*-values are emboldened. Abbreviations: SPC, secondary primary cancer; APC, annual rate of percent change, and CI, confidence interval.

Supplemental Table S5. Temporal trends for secondary primary cancer in long-term follow-up cases (for Figure 3A).

Cohort	Follow-up	Segment	Lower Endpoint	Upper Endpoint	APC	Lower CI	Upper CI	Test Statistic	*P*-value
Uterine	≥5yr	1	1973	1981	0.8	-2.5	4.3	0.5	0.63
		2	1981	1995	-3.1	-4.7	-1.4	-3.8	**<0.001**
		3	1995	2008	-10.5	-12.3	-8.7	-11.4	**<0.001**
	≥10yr	1	1973	1982	0.8	-2.1	3.8	0.6	0.57
		2	1982	1995	-4.1	-6.0	-2.1	-4.2	**<0.001**
		3	1995	2003	-10.8	-14.5	-7	-5.7	**<0.001**
Colorectal	≥5yr	1	1973	2001	-2.2	-2.8	-1.5	-7.1	**<0.001**
		2	2001	2008	-11	-14.4	-7.4	-6	**<0.001**
	≥10yr	1	1973	2003	-2.6	-3.2	-2	-8.9	**<0.001**

Significant *P*-values are emboldened. Abbreviations: yr, years; SPC, secondary primary cancer; APC, annual rate of percent change, and CI, confidence interval.

Supplemental Table S6. Temporal trends of SIR for secondary primary cancer (for Figure 3B).

Cohort	Follow-up	Segment	Lower Endpoint	Upper Endpoint	APC	Lower CI	Upper CI	Test Statistic	*P*-value
Colorectal	≥5yr	1	1975	1986	0.6	0.1	1.2	2.3	**0.027**
		2	1986	2002	-0.9	-1.2	-0.6	-6.5	**<0.001**
		3	200	2008	-3.5	-4.8	-2.2	-5.3	**<0.001**
	≥10yr	1	1975	1986	0.6	-0.2	1.3	1.6	0.12
		2	1986	2003	-1.0	-1.4	-0.7	-5.9	**<0.001**
Uterine	≥5yr	1	1975	1995	-0.4	-0.7	-0.1	-3.2	**0.003**
		2	1995	2008	-2.8	-3.2	-2.3	-12.4	**<0.001**
	≥10yr	1	1975	1995	-0.6	-0.8	-0.3	-4.5	**<0.001**
		2	1995	2003	-2.9	-3.9	-2.0	-6.2	**<0.001**

Significant *P*-values are emboldened. Abbreviations: yr, years; SPC, secondary primary cancer; APC, annual rate of percent change, and CI, confidence interval.

Supplemental Table S7. Temporal trends for secondary primary cancer per SPC timing (for Supplemental Figure S3).

Cohort	SPC Timing	Segment	Lower Endpoint	Upper Endpoint	APC	Lower CI	Upper CI	Test Statistic	*P*-value
Uterine	<5yr	1	1973	2003	0.7	-0.5	1.9	1.3	0.22
	≥5yr	1	1973	1981	1.6	-2.3	5.6	0.8	0.41
		2	1981	1995	-4.3	-6.2	-2.5	-4.7	**<0.001**
		3	1995	2003	-14.5	-18.7	-10.1	-6.4	**<0.001**
Colorectal	<5yr	1	1973	2003	-1.0	-2.1	0.2	-1.7	0.09
	≥5yr	1	1973	1984	1.4	-2.4	5.4	0.8	0.45
		2	1984	2003	-4.8	-5.9	-3.6	-8.5	**<0.001**

Significant *P*-values are emboldened. Abbreviations: yr, years; SPC, secondary primary cancer; APC, annual rate of percent change, and CI, confidence interval.

Supplemental Table S8. Temporal trends for secondary primary cancer per age (for Figure 4A-B).

Cohort	SPC Timing	Segment	Lower Endpoint	Upper Endpoint	APC	Lower CI	Upper CI	Test Statistic	*P*-value
uterine	Any timing	1	30	34	45.9	0.5	112.0	2	**0.047**
		2	34	60	0.0	-0.7	0.8	0.1	0.96
		3	60	66	6.2	1.7	10.9	2.8	**0.007**
		4	66	95	1.1	0.6	1.6	4.5	**<0.001**
	Antecedent	1	30	60	1.7	0.3	3.2	2.4	**0.021**
		2	60	88	6.1	5.4	6.8	19.1	**<0.001**
		3	88	95	-2.6	-9.9	5.4	-0.7	0.51
	Synchronous	1	36	54	-1.6	-5.9	2.9	-0.7	0.47
		2	54	95	4.2	3.3	5.1	9.4	**<0.001**
	Postcedent	1	30	34	43.4	-14.9	141.5	1.4	0.17
		2	34	75	0.9	0.4	1.3	3.7	**<0.001**
		3	75	95	-5.3	-7.7	-2.8	-4.2	**<0.001**
Colorectal	Any timing	1	30	35	19.5	-2.9	47	1.7	0.09
		2	35	66	1.5	0.9	2.0	5.7	**<0.001**
		3	66	83	-1.4	-2.1	-0.7	-4.1	**<0.001**
		4	83	95	-4.7	-6.5	-2.9	-5.1	**<0.001**
	Antecedent	1	36	57	5.4	3.1	7.8	4.7	**<0.001**
		2	57	79	1.6	0.8	2.4	4.1	**<0.001**
		3	79	95	-2.4	-3.8	-0.9	-3.3	**0.002**
	Synchronous	1	36	66	1.0	-0.7	2.7	1.2	0.23
		2	66	95	-3.1	-4.6	-1.6	-4.2	**<0.001**
	Postcedent	1	30	65	-0.6	-1.2	0.1	-1.6	0.11
		2	65	78	-4.7	-6.7	-2.8	-4.8	**<0.001**
		3	78	95	-10.4	-13.3	-7.4	-6.8	**<0.001**

Significant *P*-values are emboldened. Abbreviations: APC, annual rate of percent change, and CI, confidence interval.

Supplemental Table S9. Temporal trends for secondary primary cancer-specific survival among survivors (for Supplemental Figure S6).

Cohort	Follow-up	Segment	Lower Endpoint	Upper Endpoint	APC	Lower CI	Upper CI	Test Statistic	*P*-value
Colorectal	≥5yr	1	1973	1994	-2	-4.6	0.8	-1.5	0.15
		2	1994	2006	-12	-18	-5.6	-3.7	**<0.001**
	≥10yr	1	1973	1984	-4	-10.2	2.6	-1.4	0.20
		1	1986	1997	-8.6	-16.3	-0.1	-2.3	**0.047**
Uterine	≥5yr	1	1973	1982	0.1	-3.3	3.7	0.1	0.95
		2	1982	2001	-6.6	-7.9	-5.2	-9.4	**<0.001**
		3	2001	2008	-29.9	-37.7	-21.2	-6.2	**<0.001**
	≥10yr	1	1973	1981	-0.6	-5.8	4.8	-0.2	0.81
		2	1981	1995	-6.8	-9.6	-3.8	-4.7	**<0.001**
		3	1995	2003	-21.1	-29.3	-12	-4.5	**<0.001**

Significant *P*-values are emboldened. Abbreviations: APC, annual rate of percent change, and CI, confidence interval.

Supplemental Table S10. Temporal trends for secondary colorectal cancer among endometrial cancer (for Supplemental Figure S7A).

SPC Timing	Segment	Lower Endpoint	Upper Endpoint	APC	Lower CI	Upper CI	Test Statistic	*P*-value
Any timing	1	1973	1992	-0.4	-1.2	0.5	-0.9	0.38
	2	1992	2013	-5.5	-6.1	-5.0	-19.0	**<0.001**
Antecedent	1	1973	1990	11.1	6.6	15.9	5.1	**<0.001**
	2	1990	2001	-5.7	-10.3	-0.8	-2.4	**0.024**
	3	2001	2013	3.0	0.5	5.6	2.4	**0.021**
Synchronous	1	1973	1978	35.2	-2.4	87.1	1.9	0.07
	2	1978	2013	-1.5	-2.4	-0.7	-3.5	**0.001**
Postcedent	1	1973	1995	-2.3	-3.0	-1.5	-6.0	**<0.001**
	2	1995	2009	-9.3	-11.1	-7.5	-10.1	**<0.001**
	3	2009	2013	-38.2	-52.1	-20.3	-3.8	**<0.001**

Significant *P*-values are emboldened. Abbreviations: APC, annual rate of percent change, and CI, confidence interval.

Supplemental Table S11. Temporal trends for postcedent colorectal cancer in long-term follow-up cases with endometrial cancer (for Supplemental Figure S7B).

Follow-up	Segment	Lower Endpoint	Upper Endpoint	APC	Lower CI	Upper CI	Test Statistic	*P*-value
≥5yr	1	1973	1981	1.0	-2.4	4.6	0.6	0.56
	2	1981	1995	-3.2	-4.8	-1.5	-3.9	**<0.001**
	3	1995	2008	-10.5	-12.3	-8.7	-11.2	**<0.001**
≥10yr	1	1973	1981	1.6	-2.1	5.3	0.9	0.39
	2	1981	1995	-4.0	-5.7	-2.2	-4.7	**<0.001**
	3	1995	2003	-10.8	-14.5	-6.9	-5.5	**<0.001**

Significant *P*-values are emboldened. Abbreviations: APC, annual rate of percent change, and CI, confidence interval.

Supplemental Table S12. Temporal trends for secondary colorectal cancer among endometrioid type endometrial cancer (for Supplemental Figure S8A).

SPC Timing	Segment	Lower Endpoint	Upper Endpoint	APC	Lower CI	Upper CI	Test Statistic	*P*-value
Any timing	1	1973	1994	-0.8	-1.4	-0.1	-2.4	**0.023**
	2	1994	2013	-6.3	-7.0	-5.7	-18.4	**<0.001**
Antecedent	1	1973	1990	10.9	6.1	16	4.7	**<0.001**
	2	1990	2001	-6.2	-11.0	-1.2	-2.5	**0.018**
	3	2001	2013	2.5	-0.3	5.3	1.8	0.08
Synchronous	1	1973	2013	-0.7	-1.7	0.2	-1.6	0.11
Postcedent	1	1973	1999	-2.8	-3.4	-2.1	-8.4	**<0.001**
	2	1999	2010	-12.2	-14.7	-9.7	-9.2	**<0.001**
	3	2010	2013	-46.6	-68.8	-8.4	-2.4	**0.024**

Significant *P*-values are emboldened. Abbreviations: APC, annual rate of percent change, and CI, confidence interval.

Supplemental Table S13. Temporal trends for postcedent colorectal cancer in long-term follow-up cases with endometrioid type endometrial cancer (for Supplemental Figure S8B).

Follow-up	Segment	Lower Endpoint	Upper Endpoint	APC	Lower CI	Upper CI	Test Statistic	*P*-value
≥5yr	1	1973	1982	1.1	-2	4.3	0.7	0.48
	2	1982	1999	-4.4	-5.7	-3.0	-6.5	**<0.001**
	3	1999	2008	-12.6	-15.4	-9.7	-8.4	**<0.001**
≥10yr	1	1973	1983	1.4	-1.0	3.8	1.2	0.24
	2	1983	1990	-8.5	-13.9	-2.8	-3.1	**0.006**
	3	1990	1993	8.1	-25.3	56.6	0.4	0.66
	4	1993	2003	-10.1	-12.4	-7.8	-8.7	**<0.001**

Significant *P*-values are emboldened. Abbreviations: APC, annual rate of percent change, and CI, confidence interval.
